# Pyrotinib alone or in combination with docetaxel in refractory HER2‐positive gastric cancer: A dose‐escalation phase I study

**DOI:** 10.1002/cam4.5830

**Published:** 2023-04-20

**Authors:** Dan Liu, Furong Kou, Jifang Gong, Zhiqiang Wang, Xiaotian Zhang, Jian Li, Yan Li, Jie Li, Jun Zhou, Ming Lu, Xicheng Wang, Zhihao Lu, Yanshuo Cao, Jianjun Zou, Xiaoyu Zhu, Ruihua Xu, Lin Shen

**Affiliations:** ^1^ Key Laboratory of Carcinogenesis and Translational Research (Ministry of Education), Early Drug Development Center Peking University Cancer Hospital & Institute Beijing China; ^2^ Key Laboratory of Carcinogenesis and Translational Research (Ministry of Education), Department of Day Oncology Unit Peking University Cancer Hospital & Institute Beijing China; ^3^ Key Laboratory of Carcinogenesis and Translational Research (Ministry of Education), Department of Gastrointestinal Oncology/Early Drug Development Center Peking University Cancer Hospital & Institute Beijing China; ^4^ Department of Medical Oncology, State Key Laboratory of Oncology in South China, Collaborative Innovation Center of Cancer Medicine Sun Yat‐sen University Cancer Center Guangzhou P. R. China; ^5^ Key Laboratory of Carcinogenesis and Translational Research (Ministry of Education), Department of Gastrointestinal Oncology Peking University Cancer Hospital & Institute Beijing China; ^6^ Jiangsu Hengrui Medicine Co., Ltd Lianyungang China

**Keywords:** docetaxel, gastric cancer, HER2, phase I study, pyrotinib

## Abstract

**Aim:**

Pyrotinib (an irreversible pan‐ErbB small‐molecular tyrosine kinase inhibitor) was approved in human epidermal growth factor receptor 2 (HER2)‐positive breast cancer and showed great antitumor activity in preclinical studies of gastric cancer (GC). This study was first designed to prospectively assess pyrotinib in pretreated HER2‐positive GC.

**Methods:**

This multicenter, phase I study followed a standard “3 + 3” design and included two parts. In the pyrotinib part, pyrotinib was administered orally, once per day at dose levels of 240, 320, 400, and 480 mg. In the pyrotinib plus docetaxel part, patients received pyrotinib (qd, d1‐21, q3W) combined with docetaxel (60 mg/m^2^, d1, q3W) at dose levels of 240, 320, and 400 mg. Primary endpoints were to determine the maximum tolerated dose (MTD) and recommended phase II dose (RP2D) of pyrotinib as monotherapy or coadministered with docetaxel.

**Results:**

A total of 25 patients were enrolled and received pyrotinib (*n* = 15) or pyrotinib plus docetaxel (*n* = 10). One DLT was observed in pyrotinib monotherapy part (Grade 3 uncontrolled diarrhea after supportive care) and pyrotinib plus docetaxel part (Grade 4 neutropenia and leukopenia). In the pyrotinib monotherapy part, MTD was not reached. Diarrhea, anemia, neutropenia, and leukopenia were the most common treatment‐related adverse events (TRAEs). The RP2D for pyrotinib monotherapy was recommended as 400 mg. After combining with docetaxel, the risk of leukopenia and neutropenia was increased. Grade ≥3 TRAEs were reported for four patients in the monotherapy part and for eight patients in the combination part. Mean *t*_1/2_ was approximately 20 h. Pyrotinib exposure was dose‐dependent with a nonlinear relationship versus dose. There were five patients who had confirmed partial response (monotherapy: one each at 240, 400, and 480 mg dose cohort; combination therapy: two at 240 mg dose cohort), resulting in an objective response rate of 21% and 20%, respectively.

**Conclusions:**

Pyrotinib alone and combined with docetaxel showed acceptable toxicities in patients with pretreated HER2‐positive GC.

**Trial Registration:**

This study was registered with ClinicalTrials.gov, NCT02378389.

## Graphical abstract

This phase 1 study demonstrate the safety and efficacy of pyrotinib in HER2 positive GC. Pyrotinib alone and plus docetaxel are well‐tolerated and show anti‐tumor activity. But combination with docetaxel cannot improve the efficacy.

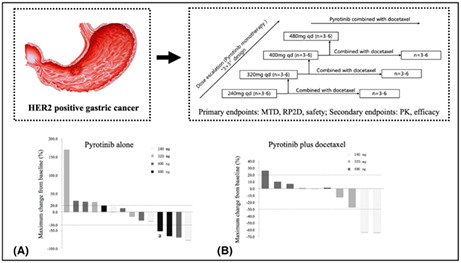

## 1 BACKGROUND

Human epidermal growth factor receptor 2 (HER2) was a key therapeutic target in gastric cancer (GC).^1^ Anti‐HER2 antibodies and HER2‐directed antibody‐drug conjugates showed great survival improvement in advanced or metastatic HER2‐positive GC.^2^ However, anti‐HER2 therapies were still limited for HER2‐positive GC patients. Only trastuzumab, disitamab vedotin (RC48), and trastuzumab deruxtecan (DS8201) have been approved and on market.^3^, ^4^, ^5^ Tyrosine kinase inhibitors (TKIs) targeting HER2 were also explored in advanced HER2‐positive GC. Lapatinib, a TKI reversibly blocking EGFR and HER2, was explored in HER2‐positive GC by LOGiC and TyTAN trial ending with negative results.^6^, ^7^ Hence, novel anti‐HER2 agents are urgently needed.

Pyrotinib was a novel oral, irreversible pan‐ErbB small‐molecular TKI blocking HER1, HER2, and HER4, which was approved in HER2‐positive breast cancer (BC). And it has been reported that pyrotinib could inhibit the proliferation of gastric cells overexpressing HER2 (NCI‐N87 and BT474 cell line) in vitro.^8^ Although a phase I trial demonstrated that pyrotinib at 400 mg had well‐tolerated toxicity and promising efficacy in patients with HER2‐positive metastatic BC,^9^ rare safety and efficacy data of pyrotinib in HER2‐positive GC was ever reported. Considering the high heterogenicity of GC, pyrotinib monotherapy might contribute to limited efficacy. To further improve the efficacy, pyrotinib in combination with chemotherapy would also be evaluated.

Docetaxel, a broad‐spectrum phase M cycle‐specific cytotoxic drug which promotes the polymerization of tubules into stable microtubules, inhibits their polymerization and significantly reduces the tumor proliferation.^10^ It was generally used in refractory GC alone and in combination with target drugs or other cytotoxic drugs as salvage therapies.^11^, ^12^ Thus, docetaxel was chosen as a combined medication with pyrotinib in this study.

Hereby, we first performed a multicenter, phase I study to prospectively assess the safety and pharmacokinetic (PK) characteristics of pyrotinib alone or plus docetaxel in refractory HER2‐positive GC patients, and explore the preliminary antitumor activity as well.

## 2 PATIENTS AND METHODS

### 2.1 Patient eligibility

Patients with pathologically confirmed advanced HER2‐positive (3+ or 2+ staining intensity by immunohistochemistry or gene amplification by fluorescence in situ hybridization amplification [HER2:CEP17 ratio ≥2]) GC who suffered previous standard treatments (without docetaxel or other anti‐HER2 TKIs) failure or intolerance were considered. Other key inclusion criteria were age 18–70 years, measurable lesions per RECIST V1.1, an Eastern Cooperative Oncology Group (ECOG) performance status of 0 or 1, adequate organ and bone marrow function, and a life expectancy of at least 3 months.

Patients were excluded if they had a history of autoimmune diseases or requirement of long‐term use of steroids (≥50 days); brain metastases; symptomatic or uncontrolled third space effusion; unable to take the drugs orally; uncontrolled hypokalemia and hypomagnesemia; a history of other antitumor therapy within 4 weeks before or during study; active hemorrhage within 2 months before study; chronic disease or gastrointestinal obstruction effecting absorption; other malignancies (except for cured locally cancers) within 5 years before study entry; a history of active hepatitis B virus, hepatitis C virus, syphilis or human immunodeficiency virus infection; uncontrolled heart disease. Each patient provided written informed consent.

### 2.2 Study design and treatment

This multicenter, open‐label, phase I dose escalation study was conducted in China from September 2014 (ClinicalTrials. gov, NCT02378389). The study was approved by the ethics committee of each study center and performed in accordance with the International Conference on Harmonization Guidelines for Good Clinical Practice and Declaration of Helsinki.

**FIGURE 1:**
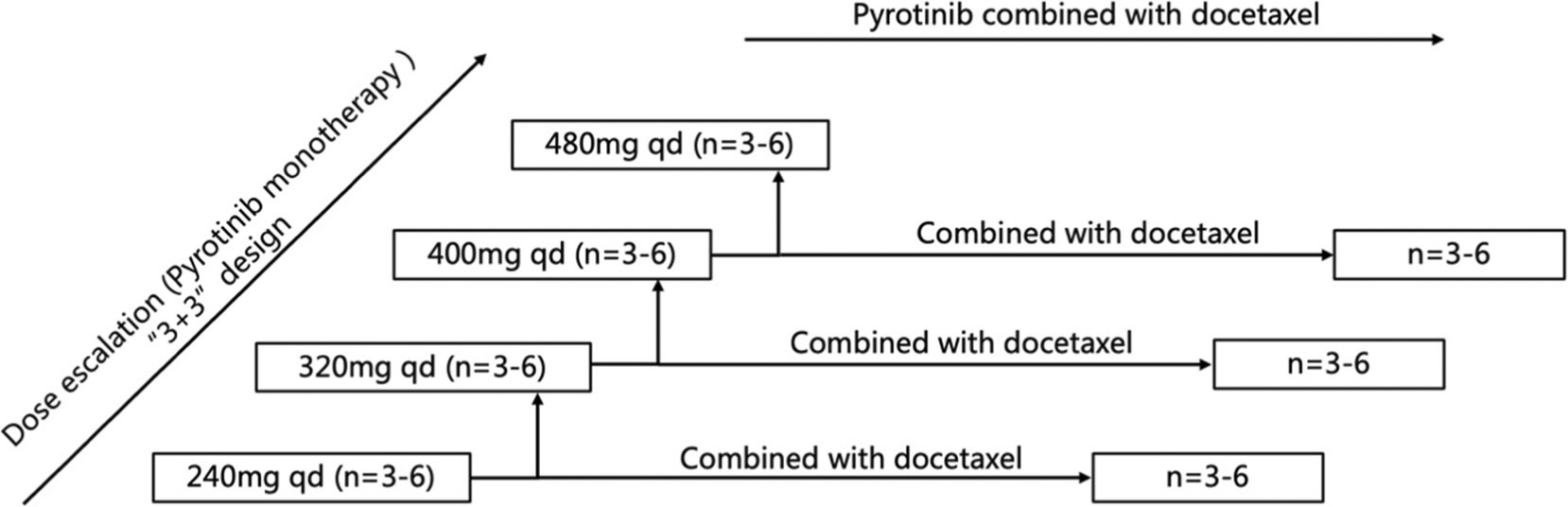
Study design.

The phase I study was composed of two parts: dose escalation of pyrotinib monotherapy and in combination with docetaxel. This study followed a standard “3+3” design (Figure 1). In the pyrotinib monotherapy part, four dose levels (240, 320, 400, and 480 mg qd, d1‐21; q3W) were designed for evaluation. Based on the findings of the pyrotinib monotherapy part, one–three dose levels would be selected for combination with docetaxel (60 mg/m^2^ d1; q3W). All patients were orally administered once‐daily continuous doses of pyrotinib within 30 min after breakfast in 21‐day cycles. In pyrotinib combined with docetaxel part, patients also received docetaxel injection via a 60 min infusion. Patients could receive study drugs until confirmed disease progression, death, intolerable toxicity, loss to follow‐up or withdrawal of informed consent. In all, 21–42 patients would be enrolled.

### 2.3 Endpoints

The primary endpoints were to assess the maximum tolerated dose (MTD), safety and the recommended phase II dose (RP2D) of pyrotinib monotherapy and pyrotinib in combination with docetaxel. The secondary endpoints included PK evaluation and preliminary efficacy of pyrotinib monotherapy and pyrotinib combined with docetaxel.

### 2.4 Safety assessment

Safety was evaluated throughout the study. All adverse events (AEs) were recorded, the severity was assessed according to NCI‐CTCAE (V.4.0), and the relationship with pyrotinib or docetaxel was judged.

DLT criteria included Grade 4 neutropenia with a duration of ≥7 days, Grade 3 or 4 neutropenia with fever, Grade 3 thrombocytopenia with bleeding tendency or Grade 4 thrombocytopenia, Grade 3 or 4 anemia; ≥ Grade 2 heart and renal insufficiency, Grade 3 or 4 non‐hematologic AE (except for Grade 3 fatigue lasting ≤3 days, recoverable diarrhea, nausea, and vomiting after supportive care). DLTs were assessed in 21 days following the first dose.

### 2.5 PK evaluation

The main PK parameters were estimated by a standard noncompartmental method using Phoenix WinNonLin (Pharsight, version 6.3, Pharsight Corp.), including elimination half‐life (*t*_1/2_) and area under the concentration‐time curve from time zero to the last measured time point (AUC_0–*t*_). Validated liquid chromatography–tandem mass spectrometry method was used to measure plasma concentrations (linearity range, 1–200 ng/mL). The peak plasma concentration (*C*_max_) and time to *C*_max_ (*t*_max_) were directly obtained from the measured concentration.

In pyrotinib monotherapy part, blood samples were obtained on D1 and D21 of the first cycle at predose; 1, 2, 3, 4, 5, 6, 8, 12, and 24 h after the first dose; and on C1D8 and C1D15 at predose. In pyrotinib in combination with docetaxel part, the samples were obtained on D1 of the first cycle at predose; 5 min, 30 min, 1, 1.25, 1.5, 2, 3, 4, 5, 6, 8, 12, and 24 h after the first dose; and on C1D8 and C1D15 at predose. Samples were also collected on Day 21 of the first cycle at predose and 1, 2, 3, 4, 5, 6, 8, 12, and 24 h after the first dose. The whole blood was centrifuged (3500 rpm at 4°C for 10 min) and obtained plasma samples were stored at −80°C until analysis.

### 2.6 Efficacy assessment

Tumor responses were evaluated based on RECIST 1.1 at screening and every two cycles until disease progression or the start of new antitumor treatment. The complete and partial response would be confirmed after 4 weeks. All the progression‐free survival (PFS) was defined as the time from the start of the first dose to disease progression or death of any cause. The overall survival was defined as the time from the start of the first dose to death. Survival status was obtained from medical records and followed up by telephone.

### 2.7 Statistical analysis

Statistical analysis was performed by SPSS Version 22.0 (SPSS Inc.). The safety was assessed in patients who received at least one dose of study drugs. Patient characteristics and efficacy were assessed in the full analysis set, including patients who received at least one dose of study drugs. Descriptive statistics were used to assess demographic characteristics, safety, and tumor response outcomes. The Kaplan–Meier method was used for time‐to‐event endpoints.

## 3 RESULTS

### 3.1 Patient characteristics

Between September 2014 and February 2017, 25 patients with HER2‐positive GC were finally enrolled in this study. The median age was 58 years (range 39–65). And the majority of patients (*n* = 21, 84%) were male. The baseline characteristics of enrolled patients are detailed in Table 1.

**TABLE 1:** Patient demographics and baseline characteristics.

Characteristics	Pyrotinib, *n* = 15	Pyrotinib+docetaxel, *n* = 10	Total, *n* = 25
Median age, years (range)	58 (43–65)	57 (39–65)	58 (39–65)
Sex			
Male	12(80.0)	9 (90.0)	21 (84.0)
Female	3(20.0)	1 (10.0)	4 (16.0)
ECOG			
0	3(20.0)	4 (40.0)	7 (28.0)
1	12(80.0)	6 (60.0)	18 (72.0)
Prior therapies			
Chemotherapy	15 (100.0)	10 (100.0)	25 (100.0)
Target therapy (except anti‐HER2)	1 (6.7)	0	1(4.0)
Radiotherapy	7 (46.7)	1 (10.0)	8 (32.0)
Surgery of gastrectomy	8 (53.3)	6 (60.0)	14 (56.9)
No. of prior anticancer regimens			
<3 lines	5(33.3)	10 (100.0)	15 (60.0)
≥3 lines	10(66.7)	0	10 (40.0)
Prior anti‐HER2 therapy			
Trastuzumab	7 (46.7)	8 (80.0)	15 (60.0)
Other anti‐HER2 therapies^a^	5 (33.3)	1 (10.0)	6 (24.0)
No	7 (46.7)	2 (20.0)	9(36.0)

*Note*: Values are expressed as *n* (%), unless otherwise stated.

^a^ Other anti‐HER2 therapies: trastuzumab emtansine (T‐DM1), pertuzumab.

All the patients (*n* = 25) received prior chemotherapies, and 10 patients (40.0%) had ≥3rd‐line treatment. More than half of the patients had surgery of gastrectomy. As for anti‐HER2 treatment history, 15 patients (60.0%) received prior treatment of trastuzumab, and 6 patients received other anti‐HER2 therapies including trastuzumab emtansine (T‐DM1) and pertuzumab. In pyrotinib monotherapy part, a total of 15 patients received one of four dose levels of pyrotinib (240 mg [*n* = 3], 320 mg [*n* = 3], 400 mg [*n* = 6], 480 mg [*n* = 3]). Another 10 patients received pyrotinib combined with docetaxel at one of three dose levels (240 mg [*n* = 3], 320 mg [*n* = 3], 400 mg [*n* = 4]).

### 3.2 Safety

A total of 25 patients were included in safety analysis (Table 2a, 2b). All patients experienced treatment‐related adverse events (TRAEs) of any grade. In the pyrotinib monotherapy part, MTD was not reached. Diarrhea (*n* = 12, 80%), anemia (*n* = 5, 33%), leukopenia (*n* = 4, 27%), and neutropenia (*n* = 4, 27%) were most commonly observed. Diarrhea was the most common grade ≥3 TRAE (*n* = 2). One of them (Grade 3) discontinued pyrotinib treatment for uncontrolled diarrhea after supportive care, which was defined as DLT at a dose level of 400 mg. Grade 3 neutropenia, transaminase, and bilirubin were also observed at a dose level of 400 and 320 mg.

**TABLE 2a:** Treatment‐related adverse events of all grades in pyrotinib monotherapy part.

Adverse event	240 mg, *n* = 3		320 mg, *n* = 3		400 mg, *n* = 6		480 mg, *n* = 3		Total, *N* = 15	
	All grade *n* (%)	G3‐4 *n* (%)	All grade *n* (%)	G3‐4 *n* (%)	All grade *n* (%)	G3‐4 *n* (%)	All grade *n* (%)	G3‐4 *n* (%)	All grade *n*(%)	G3‐4 *n*(%)
Hematologic toxicity										
Leukopenia	0	0	2 (67)	0	2 (33)	0	0	0	4 (27)	0
Neutropenia	1 (33)	0	2 (67)	0	1 (17)	1 (17)	0	0	4 (27)	1 (7)
Anemia	1 (33)	0	1 (33)	0	2 (33)	0	1 (33)	0	5 (33)	0
Thrombocytopenia	0	0	0	0	1 (17)	0	0	0	1 (7)	0
Non‐hematologic toxicity										
Diarrhea	2 (67)	0	3 (100)	0	5 (83)	2 (33)	2 (67)	0	12 (80)	2 (13)
Increased bilirubin	2 (67)	0	1 (33)	1(33)	0	0	0	0	3 (20)	1 (7)
Fatigue	1 (33)	0	1 (33)	0	1 (17)	0	0	0	3 (20)	0
Nausea	1 (33)	0	0	0	2 (33)	0	0	0	3 (20)	0
Anorexia	1 (33)	0	0	0	2 (33)	0	0	0	3 (20)	0
Acid regurgitation	1 (33)	0	1 (33)	0	1 (17)	0	0	0	3 (20)	0
Abdominal discomfort	0	0	1 (33)	0	2 (33)	0	0	0	3 (20)	0
Abdominal pain	0	0	0	0	2 (33)	0	0	0	2 (13)	0
Rash	0	0	1 (33)	0	0	0	1 (33)	0	2 (13)	0
Heartburn	0	0	1 (33)	0	1 (17)	0	0	0	2 (13)	0
Increased transaminase	0	0	1 (33)	1 (33)	0	0	0	0	1 (7)	1 (7)
Increased creatinine	0	0	0	0	1 (17)	0	0	0	1 (7)	0
Cutaneous pruritus	0	0	0	0	1 (17)	0	0	0	1 (7)	0
Weight loss	0	0	0	0	1 (17)	0	0	0	1 (7)	0
Hematochezia	0	0	1 (33)	0	0	0	0	0	1 (7)	0
Fullness in head	1 (33)	0	0	0	0	0	0	0	1 (7)	0
Dysgeusia	0	0	0	0	1 (17)	0	0	0	1 (7)	0

*Note*: Values are expressed as *n* (%), unless otherwise stated.

**TABLE 2b:** Treatment‐related adverse events of all grades in pyrotinib combined with docetaxel part.

Adverse event	240 mg, *n* = 3		320 mg, *n* = 3		400 mg, *n* = 4		Total, *N* = 10	
	All grade	G3‐4	All grade	G3‐4	All grade	G3‐4	All grade	G3‐4
Hematologic toxicity								
Leukopenia	2 (67)	2 (67)	3 (100)	2 (67)	4 (100)	2 (50)	9 (90)	6 (60)
Neutropenia	2 (67)	2 (67)	3 (100)	2 (67)	4 (100)	3 (75)	9 (90)	7 (70)
Anemia	2 (67)	0	3 (100)	0	3 (75)	1 (25)	8 (80)	1 (10)
Thrombocytopenia	0	0	1 (33)	0	1 (25)	0	2 (20)	0
Non‐hematologic toxicity								
Diarrhea	2 (67)	0	2 (67)	1 (33)	2 (50)	0	6 (60)	1 (10)
Increased transaminase	2 (67)	0	0	0	2 (50)	1 (25)	4 (40)	1 (10)
Neurotoxicity	2 (67)	0	1 (33)	0	0	0	3 (30)	0
Increased bilirubin	0	0	1 (33)	0	2 (50)	0	3 (30)	0
Fatigue	0	0	1 (33)	0	1 (25)	0	2 (20)	0
Oral ulcer	1 (33)	0	1 (33)	0	0	0	2 (20)	0
Nausea	1 (33)	0	1 (33)	0	0	0	2 (20)	0
Vomiting	1 (33)	0	0	0	0	0	1 (10)	0
Anorexia	0	0	1 (33)	0	0	0	1 (10)	0
Acid regurgitation	0	0	1 (33)	0	0	0	1 (10)	0
Rash	0	0	1 (33)	0	0	0	1 (10)	0
Cutaneous pruritus	1 (33)	0	0	0	0	0	1 (10)	0
Alopecia	1 (33)	0	0	0	0	0	1 (10)	0
Muscle and joint pain	0	0	1 (33)	0	0	0	1 (10)	0
Hand foot syndrome	1 (33)	0	0	0	0	0	1 (10)	0
Weight loss	0	0	1 (33)	0	0	0	1 (10)	0
Infusion reaction	0	0	1 (33)	0	0	0	1 (10)	0

In the combination with docetaxel part, for no DLT observation of pyrotinib alone at dose level of 240 mg and occurrence of effective case (*n* = 1), we set the initial dose of pyrotinib at 240 mg for combination with docetaxel. One DLT (Grade 4 neutropenia and leukopenia) was observed in patients received pyrotinib at 400 mg plus docetaxel. The majority of patients suffered hematologic toxicities including leukopenia (*n* = 9, 90%), neutropenia (*n* = 9, 90%), and anemia (*n* = 8, 80%). The most common non‐hematologic toxicity was diarrhea (6/10, 60%), which is similar to the results in the monotherapy part. Neutropenia and leukopenia were the most common ≥3 TRAEs.

### 3.3 PK

**FIGURE 2:**
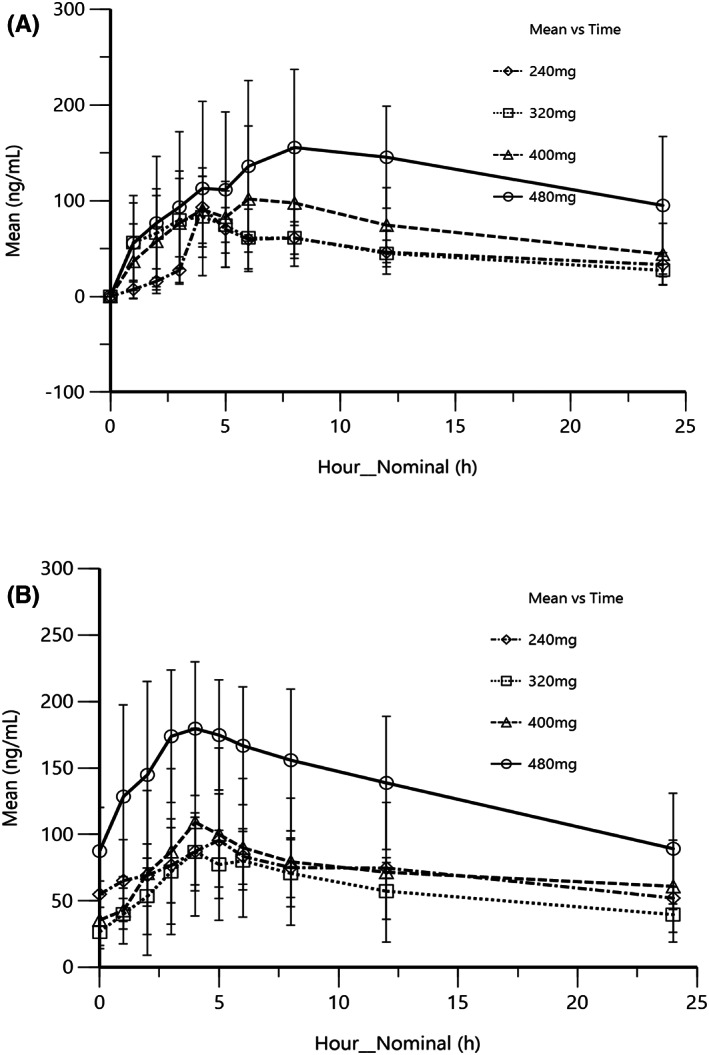
Mean plasma concentration of pyrotinib following (A) single and (B) multiple doses in the monotherapy part (PK population).

Totally, 22 patients provided plasma samples for PK analysis (monotherapy, *n* = 15; combination therapy, *n* = 7). The serum concentrations and PK data are shown in Tables S1 and S2 and in Figure 2. After single dose of pyrotinib monotherapy, the median *t*_1/2_ was approximately 20 h (14–34 h). A linear regression model was adopted to assess the relationship between dose and exposure at steady state. The result indicated that *C*_max_ and AUC_0–24_ did not increase in proportion to pyrotinib doses, which suggested a nonlinear relationship for *C*_max_ and AUC_0–24_ versus dose. Besides, there were large variations in the ratio of combination therapy to monotherapy for *C*_max_ and AUC_0–24_ of pyrotinib.

### 3.4 Antitumor activity

**FIGURE 3:**
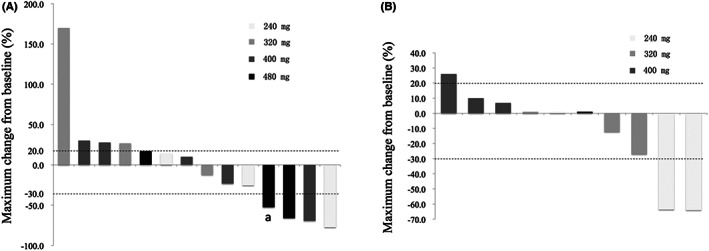
Waterfall plot of best objective response of all enrolled patients. Maximum reduction in target lesions from baseline for patients in the 240–480 mg dose cohorts in (A) the monotherapy part and (B) the combination therapy part. The best response for target lesions per patient was determined on the basis of RECIST 1.1 criteria. Dashed lines at −30 and 20 indicate RECIST 1.1 criteria for partial response and progressive disease, respectively. ^a^One patient had a 52.6% reduction in target lesion size after two cycles of treatment, but had progressive disease with partial bowel obstruction after four cycles of treatment. So, the confirmed overall response was therefore classified as stable disease.

In all, from September 2014 to February 2017, 24 patients finished efficacy assessment (monotherapy, *n* = 14; combination therapy, *n* = 10). One patient at 400 mg dose level of pyrotinib monotherapy part withdrew from the study because of DLT (Grade 3 diarrhea) before efficacy assessment. So, the patient was not evaluable. A summary of the antitumor activity per investigator is shown in Table 3 and Figure 3.

**TABLE 3:** Best overall response in evaluable population in monotherapy part and combination therapy part.

Dose cohorts	Best response				Efficacy	
	CR	PR	SD	PD	ORR, *n* (%)	DCR, *n* (%)
Monotherapy part						
240 mg (*n* = 3)	0	1	0	2	1 (33)	1 (33)
320 mg (*n* = 3)	0	0	1	2	0	1 (33)
400 mg (*n*=5^a^)	0	1	1	3	1 (20)	2 (40)
480 mg (*n* = 3)	0	1	1^b^	1	1 (33)	2 (67)
Total (*N* = 14)	0	3	3	8	3 (21)	6 (43)
Combination therapy part						
240 mg (*n* = 3)	0	2	1	0	2 (67)	3 (100)
320 mg (*n* = 3)	0	0	3	0	0	3 (100)
400 mg (*n* = 4)	0	0	1	3	0	1 (25)
Total (*N* = 10)	0	2	5	3	2 (20)	7 (70)

Abbreviations: CR, complete response; DCR, disease control rate: (CR + PR + SD) %; ORR, overall response rate: (CR + PR) %; PD, progressive disease; PR, partial response; SD, stable disease.

^a^ Six patients were enrolled in the 400 mg dose cohort. One patient was not evaluable. This patient received one cycle treatment and experienced Grade 3 diarrhea, which was defined as DLT. Finally, this patient dropped out of the study.

^b^ This patient achieved PR after two cycles of treatment, but had PD after four cycles of treatment. So, the confirmed efficacy was SD. Cut‐off date: February 28, 2017.

In the pyrotinib monotherapy part, the overall objective response rate (ORR) was 21% and the disease control rate (DCR) was 43%, with three patients achieving PR, three patients achieving stable disease, eight patients suffering progressive disease. The patients achieving PR were distributed in dose levels of 240, 400, and 480 mg. None of the patients achieving PR received prior anti‐HER2 regimens. The PFS ranged from 2.67 to 20.47 months. Of note, at the time of data cut‐off (February 28, 2017), one patient with PFS of 20.47 months was at the 400 mg dose level and was still ongoing with pyrotinib. Based on the safety data, PK analysis in this study and the results of phase I study in BC,^9^ the dose of 400 mg was considered as RP2D.

In the pyrotinib combined with docetaxel part, there were two patients achieving PR, five patients experiencing SD, with the ORR and DCR of 20% and 70%, respectively. At the dose level of 400 mg, although only one patient suffered DLT, three of four patients experienced quick disease progression. Hence, the enrollment of this dose level was stopped early for poor efficacy. Based on the data of safety, PK, and efficacy, the RP2D of pyrotinib was 400 mg.

## 4 DISCUSSION

Pyrotinib is an irreversible pan‐ErbB small‐molecular TKI, which has been approved in HER2‐positive BC. While the safety and efficacy data were limited in HER2‐positive GC, only several small‐sample retrospective studies and case reports involved it.^13^, ^14^ And the effect of chemotherapy combined with pyrotinib in GC was unknown. A study was necessary to prospectively explore the safety and antitumor activity in HER2‐positive GC. This multicenter, phase I, open‐label study first demonstrated that pyrotinib, both alone and combined with docetaxel, was generally well tolerated and showed comparable antitumor activity in patients with HER2‐positive advanced GC.

In the monotherapy part, diarrhea was the most common TRAE. And two patients (13%) experienced Grade 3 diarrhea in the 400 mg dose cohort, one of which was defined as DLT. In the phase I study in HER2‐positive BC, pyrotinib monotherapy was generally well tolerated at doses up to 480 mg.^9^ DLT was Grade 3 diarrhea in the 480 mg dose cohort, and MTD was 400 mg. This study showed that dose‐producing DLT was lower in GC patients compared with BC patients (400 mg vs. 480 mg). Generally, the performance status of the GC patients was poorer than that of BC patients, making it difficult to ascend to high dose level in GC patients. So, the RP2D of the pyrotinib was determined as 400 mg. Moreover, the overall spectrum of AEs observed in BC was similar to that in GC. Besides, the AEs of pyrotinib were similar to those reported for other anti‐HER2 TKIs (such as lapatinib and neratinib), with the most common AEs of diarrhea.^7^, ^15^ After combination with docetaxel, the most common TRAEs were hematologic toxicities (*n* = 9, 90%). The most common non‐hematologic toxicity was still diarrhea (*n* = 6, 60%), with one Grade 3 diarrhea (10%). The combination therapy showed a higher incidence of Grade ≥3 TRAEs compared with monotherapy (80% vs. 27%). However, we speculated that these hematologic and Grade 3 toxicities were more likely caused by docetaxel.

Pyrotinib exposure was dose‐dependent with a nonlinear relationship versus dose, which was different from the PK profile data reported in the metastatic BC population. Besides, limited data suggested coadministration with docetaxel had no apparent effect on the PK of pyrotinib.

The ORR of pyrotinib alone and pyrotinib combined with docetaxel was 21% and 20%, which was relatively unsatisfied in patients with pretreated HER2‐positive GC. Unlike BC, pyrotinib alone could not show encouraged efficacy in HER2‐positive GC. But there were indeed some patients who achieved long‐lasting disease control time for more than 12 months. So, pyrotinib might be effective in some selected patients. This similar result was also shown in studies of other anti‐HER2 TKIs, including lapatinib, afatinib, and neranib.^6^, ^16^ The underlying biological differences between HER2‐positive GC and BC might be one of the reasons, while the differences have influence on the responses to HER2‐directed therapies. GC showed more heterogeneity of HER2 expressions and incomplete membrane staining as compared with BC. Discordance in HER2 status was reported between primary and metastatic lesions, which could affect the efficacy in patients.^17^, ^18^ Besides, some HER2‐positive patients converted to HER2‐negative after failure of chemotherapy.^19^, ^20^ It reminded us of the reassessment of HER2 status after disease progression. Unfortunately, our study did not re‐evaluate HER2 status before initiating anti‐HER2 therapy. Besides, only one of five PR patients had prior anti‐HER2 regimes. For the small sample size, whether pervious exposure of trastuzumab affects the efficacy of pyrotinib warrants further evaluation. Hence, future studies should highlight the importance of confirmation of HER2 status before giving drugs targeting HER2, as well as exploring the effective biomarkers of pyrotinib and better identifying HER2‐driven GC patients who might benefit more from anti‐HER2‐targeted therapies.

The main limitations of this study are typical of early‐phase clinical studies. Most clinical cohorts had a small size and required further investigations. The lack of a control arm makes it difficult to accurately confirm the effect of combination with docetaxel. But based on the early signs demonstrated in this study, the combination with docetaxel increased the toxicity, rather than the efficacy, which might suggest us to try other combination strategy. Furthermore, the resistance mechanism of pyrotinib had been explored in cell lines and patient‐derived xenograft models. Dysregulation of the CCND1‐CDK4/6‐Rb pathway was found to be to the main cause of pyrotinib resistance in preclinical AVATAR mouse.^21^ Hence, combination pyrotinib with CDK4/6 inhibitor might improve efficacy. A clinical trial of pyrotinib in combination with CDK4/6 inhibitor has been designed and conducted (NCT03480256).

## 5 CONCLUSIONS

In brief, pyrotinib alone and combined with docetaxel had shown acceptable safety profile in HER2 positive gastric cancer. Pyrotinib plus docetaxel might not further improve the efficacy.

## AUTHOR CONTRIBUTIONS

**Dan Liu:** Conceptualization (equal); data curation (equal); formal analysis (equal); investigation (equal); project administration (equal); resources (equal); supervision (equal); validation (equal); visualization (equal); writing – original draft (lead); writing – review and editing (lead). **Furong Kou:** Conceptualization (supporting); data curation (lead); formal analysis (lead); investigation (equal); methodology (equal); project administration (equal); resources (lead); software (lead); supervision (supporting); validation (equal); visualization (equal); writing – original draft (equal); writing – review and editing (supporting). **Jifang Gong:** Conceptualization (equal); data curation (equal); formal analysis (lead); investigation (lead); project administration (lead); resources (equal); supervision (equal); validation (equal); visualization (equal); writing – original draft (supporting); writing – review and editing (supporting). **Zhiqiang Wang:** Conceptualization (equal); data curation (supporting); formal analysis (supporting); investigation (supporting); resources (supporting); software (supporting); supervision (supporting); validation (equal); visualization (equal). **Xiaotian Zhang:** Conceptualization (supporting); data curation (supporting); formal analysis (supporting); investigation (supporting); resources (supporting); supervision (supporting); validation (supporting); visualization (supporting). **Jian Li:** Conceptualization (supporting); data curation (supporting); formal analysis (supporting); investigation (supporting); resources (supporting); supervision (supporting); validation (supporting); visualization (supporting). **Yan Li:** Conceptualization (supporting); data curation (supporting); formal analysis (supporting); investigation (supporting); resources (supporting); software (supporting); supervision (supporting); validation (supporting); visualization (supporting). **Jie Li:** Conceptualization (supporting); data curation (supporting); formal analysis (supporting); investigation (supporting); resources (supporting); supervision (supporting); validation (supporting); visualization (supporting). **Jun Zhou:** Conceptualization (supporting); data curation (supporting); formal analysis (supporting); investigation (supporting); resources (supporting); supervision (supporting); validation (supporting); visualization (supporting). **Ming Lu:** Conceptualization (supporting); data curation (supporting); formal analysis (supporting); investigation (supporting); resources (supporting); supervision (supporting); validation (supporting); visualization (supporting). **Xicheng Wang:** Conceptualization (supporting); data curation (supporting); formal analysis (supporting); investigation (supporting); resources (supporting); supervision (supporting); validation (supporting); visualization (supporting). **Zhihao Lu:** Conceptualization (supporting); data curation (supporting); formal analysis (supporting); investigation (supporting); resources (supporting); supervision (supporting); validation (supporting); visualization (supporting). **Yanshuo Cao:** Conceptualization (supporting); data curation (supporting); formal analysis (supporting); investigation (supporting); resources (supporting); supervision (supporting); validation (supporting); visualization (supporting). **Jianjun Zou:** Conceptualization (supporting); data curation (supporting); formal analysis (supporting); funding acquisition (lead); methodology (lead); resources (supporting); supervision (supporting); validation (supporting); visualization (supporting). **Xiaoyu Zhu:** Conceptualization (supporting); data curation (supporting); formal analysis (supporting); project administration (supporting); resources (supporting); software (equal); supervision (supporting); validation (supporting); visualization (supporting). **Rui‐Hua Xu:** Conceptualization (lead); data curation (equal); formal analysis (supporting); investigation (lead); project administration (supporting); resources (supporting); supervision (supporting); validation (supporting); visualization (supporting). **Lin Shen:** Conceptualization (lead); data curation (equal); formal analysis (lead); funding acquisition (supporting); investigation (lead); project administration (lead); resources (lead); supervision (equal); validation (lead); visualization (equal); writing – review and editing (equal).

## FUNDING INFORMATION

This phase I clinical trial (NCT02378389) was sponsored by Jiangsu Hengrui Medicine Co., Ltd., and supported by the National Natural Science Foundation of China (no. 91959205).

## CONFLICT OF INTEREST STATEMENT

Jianjun Zou and Xiaoyu Zhu are employees of Hengrui. All other authors declare no competing interests.

## ETHICS STATEMENT

This study was approved by the ethics committee of Beijing Cancer Hospital (2014YW17) and Sun Yat‐sen University Cancer Center (A2014‐029‐02).

## Supporting information


**Table S1.** Table S2.

## Data Availability

Reasonable request for data sharing should be submitted to the corresponding author after the indication has been approved in China. The sponsor will review the proposal and consider to share the data providing the requestor signs a data‐access agreement.
