# Supplementary material for: Pyrotinib alone or in combination with docetaxel in refractory HER2‐positive gastric cancer: A dose‐escalation phase I study
Source: Cancer Med. 2023 Apr 20;12(9):10704–14. doi: 10.1002/cam4.5830 (PMC10225203; doi:10.1002/cam4.5830)
Supplement: Supplementary file 1 — Table S1. Table S2. [file CAM4-12-10704-s001.docx]

**Supplementary Materials**

**Part 1. PK analysis**

Table S1. Pharmacokinetic paramenter of pyrotinib after single and multiple doses in monotherapy part and combination part.

| **Monotherapy part** | | | | | | | | | | | | | | | |
| --- | --- | --- | --- | --- | --- | --- | --- | --- | --- | --- | --- | --- | --- | --- | --- |
| PK parameter | | Single dosing (D1) | | | | | |  | Multiple dosing (D21) | | | | | | |
|  |  | 240 mg (n=3) | 320 mg (n=3) | | 400 mg (n=6) | | 480 mg (n=3) |  | 240 mg (n=3) | | 320 mg (n=3) | | 400 mg (n=4) | | 480 mg (n=3) |
| C_max_, ng/mL | | 86(56) | 92(38) | | 108(54) | | 151(54) |  | 98(28) | | 84(37) | | 98(64) | | 179(30) |
| T_max_^a^, h | | 4(4, 4) | 4(3, 8) | | 5(3, 8) | | 8(8, 12) |  | 5(5, 12) | | 4(4, 6) | | 4(4, 6) | | 4(3, 5) |
| T_1/2_, h | | 20(34) | 14(51) | | 16(79) | | 25(175) |  | 34(76) | | 18(43) | | 16(67) | | 19(34) |
| AUC_0-inf_, ng.h/mL | | 1921(49) | 1710(50) | | 2527(75) | | 6007(259) |  | 4388(40) | | 2302(63) | | 2175(121) | | 5327(68) |
| AUC_0-24_, ng.h/mL | | 964(56) | 1120(26) | | 1461(52) | | 2432(81) |  | 1653(19) | | 1303(38) | | 1322(86) | | 3011(45) |
| CL/F, L/h | | 125(49) | 187(50) | | 158(75) | | 80(259) |  | 145(19) | | 246(38) | | 303(86) | | 159(45) |
| **Combination therapy part** | | | | | | | | | | | | | | | |
| PK parameter | Single dosing (D1) | | | | | | |  | | Multiple dosing (D21) | | | | | |
|  | 240 mg(n=2) | | | 320 mg (n=3) | | 400 mg (n=2) | |  | | 240 mg (n=3) | | 320 mg (n=3) | | 400 mg (n=1) | |
| C_max_, ng/mL | 83(42) | | | 78(60) | | 84(58) | |  | | 105(68) | | 83(80) | | 166 | |
| T_max_^a^, h | 4(4, 4) | | | 5(5, 6) | | 6(4, 8) | |  | | 4(3, 5) | | 4(1, 5) | | 1 | |
| T_1/2_, h | 6(0) | | | 9(3) | | 9(1) | |  | | 11(20) | | 37(78) | | 17 | |
| AUC_0-inf_, ng.h/mL | 599(2) | | | 1162(54) | | 1030(32) | |  | | 1383(34) | | 3512(57) | | 2825 | |
| AUC_0-24,_ng.h/mL | 547(2) | | | 929(55) | | 825(41) | |  | | 1042(40) | | 1225(60) | | 1842 | |
| CL/F, L/h | 401(2) | | | 275(54) | | 388(32) | |  | | 230(40) | | 261(60) | | 217 | |

Abbreviations: Values are presented as geometric mean (% coefficient of variation) unless otherwise stated; AUC, area under the plasma concentration–time curve; AUC_0–24_, area under the plasma concentration–time curve from time zero to 24 h; AUC_0–inf_, area under the plasma concentration–time curve from time zero to the time of the last quantifiable concentration; CL/F, total apparent drug clearance; C_max,_ maximum plasma concentration; T_1⁄2_, terminal elimination half-life; T_max_, time to reach maximum plasma concentration. ^a^T_max_ is presented as median (Min, Max).

Table S2. Comparison for pharmacokinetic parameters of pyrotinib in monotherapy and combination part

| **PK parameter** | **240 mg** | | | **320 mg** | | | **400 mg** | | |
| --- | --- | --- | --- | --- | --- | --- | --- | --- | --- |
|  | **Monotherapy** | **Combination therapy** | **Ratio^a^** | **Monotherapy** | **Combination therapy** | **Ratio^a^** | **Monotherapy** | **Combination therapy** | **Ratio^a^** |
| **Single dosing (D1)** |  |  |  |  |  |  |  |  |  |
| C_max_, ng/mL | 86(56) | 83(42) | 0.97 | 92(38) | 78(60) | 0.85 | 108(54) | 84(58) | 0.77 |
| AUC_0-24_, ng.h/mL | 964(56) | 547(2) | 0.57 | 1066(23) | 929(55) | 0.87 | 1461(52) | 825(41) | 0.56 |
| **Multiple dosing (D21)** |  |  |  |  |  |  |  |  |  |
| C_max_, ng/mL | 98(28) | 105(68) | 1.07 | 84(37) | 83(80) | 0.98 | 98(64) | 166 | 1.67 |
| AUC_0-24_, ng.h/mL | 1653(19) | 1042(40) | 0.63 | 1303(38) | 1225(60) | 0.94 | 1322(86) | 1842 | 1.39 |

Abbreviations: Values are presented as geometric mean (% coefficient of variation) unless otherwise stated; C_max_, maximum plasma concentration; AUC_0–24_, area under the plasma concentration–time curve from time zero to 24 h. ^a^Ratio of combination therapy/monotherapy
